# Cognitive and Mood Effect of Alpha-Lipoic Acid Supplementation in a Nonclinical Elder Sample: An Open-Label Pilot Study

**DOI:** 10.3390/ijerph20032358

**Published:** 2023-01-28

**Authors:** Gianpaolo Antonio Basile, Fiammetta Iannuzzo, Francesco Xerra, Giovanni Genovese, Gianluca Pandolfo, Clemente Cedro, Maria Rosaria Anna Muscatello, Antonio Bruno

**Affiliations:** 1 Department of Biomedical, Dental Sciences and Morphological and Functional Imaging, University of Messina, Via Consolare Valeria 1, Contesse, 98125 Messina, Italy; 2 Psychiatry Unit, Polyclinic Hospital University of Messina, Via Consolare Valeria 1, Contesse, 98125 Messina, Italy

**Keywords:** alpha-lipoic acid, ALA, memory, cognition, executive functions, nutraceutics

## Abstract

Background: Memory disorders are common among elder people, and nonclinical cognitive decline is commonly experienced with age. Preclinical investigations have explored the possible role of alpha-lipoic acid (ALA), a known antioxidant compound abundant in vegetables and animal tissues, in reducing oxidative stress in the aging brain and preventing cognitive decline. However, clinical evidence is limited, and the few existing results are contrasting. In addition, while most of the existing trials have been focused on the effects of ALA administration in Alzheimer’s disease (AD) or other types of dementia, studies evaluating its effects on nonclinical elder population are still missing. Methods: In the present open-label, pilot study, fifteen elder patients (mean age: 84.5 ± 5.77) received ALA at a daily dose of 600 mg/day for 12 weeks. General cognitive function, executive function, and mood symptom assessment were carried out at baseline and at the endpoint. Results: Overall, ALA administration was generally well-tolerated (only one dropout due to gastrointestinal side effects). However, no statistically significant effects either on cognitive function, executive function, or mood were found. Conclusions: Despite several limitations, our study found no evidence of positive effects on cognition and mood after ALA administration in elder people without the diagnosis of AD or cognitive impairment. Further clinical trials are needed to better investigate ALA effectiveness on cognition and mood in elder subjects.

## 1. Introduction

Aging is a physiological process that involves genetic, biological, environmental, and socioeconomic factors; it leads to a progressive, gradual deterioration of the body’s biochemical and physiological functions. Cognitive functions commonly decline with aging, and disorders involving memory, executive functions, speech, and logical reasoning ability, such as Alzheimer’s disease (AD) or mild cognitive impairment (MCI), are prevalent in the older age [1]; however, subjects who do not suffer from these conditions may also experience subclinical changes in cognitive functions while aging, sometimes affecting the general quality of life [2].

Alpha-lipoic acid (ALA), also known as thioctic acid, is a nutraceutical compound with known antioxidant properties [3]. At a cellular level, ALA works as a co-factor for multiple enzymes involved in energy metabolism and in the biosynthesis of amino acids, such as pyruvate dehydrogenase, alpha-ketoglutarate dehydrogenase, and branched-chain keto-acid dehydrogenase [4].

When introduced, a large part of ALA is reduced into dihydrolipoic acid (DHLA) by the lipoamide dehydrogenase enzyme through the involvement of the NADH/NADPH system [5]; both molecules are known to be powerful natural antioxidants [6], as they are able to interfere with reactive oxygen and nitrogen species [3,7]. In addition, both ALA and DHLA can chelate redox-active metals, such as copper, iron, or zinc, which mediate the generation of free radicals and have a potential toxic, carcinogenetic, and pro-inflammatory action [8].

Among biological factors, oxidative stress plays a major role in body aging and is thought to mediate common mechanisms underlying both physiological brain aging and pathological changes observed in MCI and AD [9].

The administration of ALA has been shown to reduce lipid peroxidation and increase the activity of antioxidant molecules in different areas of the brain of experimental animals [10,11]. In elder rats, ALA has been demonstrated to revert the effects of age-induced oxidative damage in the prefrontal cortex and hippocampi [12]. In addition, ALA has been suggested to improve memory by increasing the activity of choline acetyltransferase (ChAT), a crucial step in the biosynthesis of acetylcholine, in the hippocampi of treated rats [13]. In elder rats, dietary supplementation with ALA has also been shown to revert age-related changes in synaptic function, such as impairment of long-term potentiation and glutamate release, and to restore interleukin 1β and tocopherol levels back to their corresponding levels in young rats [14]. In vitro and in vivo investigations suggest that ALA administration can inhibit the formation of beta-amyloid fibrils and their expansion, thus exerting a direct effect on a known mechanism involved in neurodegenerative diseases [15], and that it may protect cortical neurons against amyloid-induced cell death [16].

ALA is abundantly present in vegetables and animal tissues [17], is promptly bioavailable, and has no known toxic effects on animals and human subjects [18]. Common dietary sources of ALA are meat, kidney, and liver, as well as fruits and vegetables, but the dietary intake is probably negligible; ALA absorption is in competition with other nutrients and generally is higher when assumed as a food supplement in the form of sodic salt [19]. Despite most of the ALA intake being distributed to other organs, such as the muscles, heart, and liver, ALA has been demonstrated to successfully cross the blood–brain barrier in animal models, allowing for therapeutic effects to be exerted in the central nervous system [10]. Food supplementation with ALA is safe and well-tolerated, as there is no known evidence of toxicity in animals as well as in humans, and no collateral effects have been observed at the oral daily doses currently employed as supplements (from 50 to 2400 mg/day) [20,21]. In addition, ALA supplementation has been shown to have positive systemic and metabolic effects and has been tested in multiple randomized controlled trials for the treatment of diabetes, diabetic neuropathy and retinopathy, metabolic syndrome, multiple sclerosis, as well as metabolic side effects of second-generation antipsychotics [22,23,24,25]. Taken together, all these features make ALA an ideal candidate as a nutraceutical agent to treat cognitive decline and possibly prevent neurodegeneration in older people [26]. Following this rationale, many studies have investigated cognitive changes after the administration of ALA in cognitively impaired old patients, but the resulting evidence is mixed. Earlier investigations experimented ALA as an add-on to acetylcholinesterase inhibitors; patients received a 600 mg dose of ALA for 12 months, 3 months after the standard treatment initiation [27]; follow-ups were protracted until 48 months and showed a lower disease progression rate for subjects taking ALA [28]. Another preliminary study observed improved cognitive performance and slowed down the cognitive decline in a small sample (*n* = 37) of elder patients with moderate dementia and depression after the administration of a two-week integrative treatment protocol including anti-depressants, cholinesterase inhibitors, dietary and lifestyle counseling, and supplementation of antioxidants including ALA [29]. While these preliminary works are encouraging and suggest that ALA could effectively slow down the progression of cognitive decline in patients with mild or moderate dementia, the presence of confounding factors, such as concurrent medication or combined treatment with different antioxidant molecules, lack of randomization or blinding, and reduced number of patients, limits the interpretation of the results. In addition, direct effects on memory and executive functions were not clearly demonstrated, and the few existing randomized controlled trials obtained contrasting results. Galasko and colleagues conducted a three-arm double-blind, placebo-controlled clinical trial on patients with mild to moderate Alzheimer’s disease treated with either acetylcholinesterase or memantine; the treatment included (1) high-dose ALA (900 mg/day) combined with vitamins C and E, (2) coenzyme Q, and (3) placebo. The striking result was that high-dose ALA administration in combination with vitamins C/E resulted in a faster disease progression rate [30]. On the other hand, Shinto et al. compared the efficacy of a twelve-month administration of omega-three fatty acids alone or in combination with ALA (600 mg/day) versus placebo, demonstrating a slowdown of cognitive decline in the omega-three/ALA group at the treatment termination. Notice that, in all of these randomized trials, ALA was administered in combination with other antioxidant molecules; to the best of our knowledge, randomized clinical trials evaluating the cognitive effects of ALA administration alone are completely missing. Taken together, these results underline the need for further investigations to accept or discard ALA as a possible nutraceutical therapy in memory disorders. Furthermore, while the existing works have focused on the role of ALA as an adjunctive treatment in patients suffering from dementia, studies evaluating its role on cognitively intact or subclinically impaired elder subjects are missing. It can be hypothesized that, in patients with clinically relevant cognitive impairment, the beneficial effects of ALA on memory and executive functions may be masked by the undergoing neurodegenerative process, as well as by the presence of other common psychiatric comorbidities, such as depression.

The current open-label, pilot study aims at evaluating the effects of ALA administration on cognitive and executive functioning on a sample of subclinical elder patients. Subjects were administered with an oral supplement containing ALA (600 mg/day), and the effects on memory, executive functions, and mood were evaluated after a 12-week follow-up.

## 2. Materials and Methods

### 2.1. Study Design

This was a 12-week, preliminary, uncontrolled open-label study aimed to evaluate the efficacy on cognition of the oral administration of ALA. Alpha-lipoic acid was administered at an oral daily dose of 600 mg (600 mg tablet once a day) and was maintained unchanged until the end of the study. During the study, no additional medication, including antidepressant or anxiolytic medication, was allowed. Drug administration and assessment were carried out at the Psychiatry Unit of the University Hospital of Messina, Italy. The protocol has been approved by the ethical committee of the University of Messina.

### 2.2. Subjects

A total of 15 elder subjects (8 men, 7 women) were recruited at retirement homes and older people facilities near Messina, Italy. The only inclusion criterion was age >65 years; patients were excluded if they were diagnosed with dementia, mental retardation, or concurrent major medical illnesses that could interfere with the study protocol, or if they had history of alcohol or substance abuse, head trauma, or brain lesions. Concomitant medical therapies were left untouched during the entire study period, and no additional medication, including antidepressant or anxiolytic medications, was administered during the study. All the participants gave written, informed consent prior to entering the study, and the protocol was conducted in full compliance with the Declaration of Helsinki.

### 2.3. Assessment

After recruitment, patients attended two visits: baseline (day 0) and endpoint (week 12). At each visit, patients underwent complete neuropsychological assessment. The neuropsychological assessment included the following: general cognitive screening using the Italian version of Mini Mental State Examination (MMSE) [31]; an Italian neuropsychological battery (Esame Neuropsicologico Breve, ENB2) including measures of verbal short- and long-term memory, forward digit span, visuospatial abilities (Trail Making Test—A), phonemic fluency test (letters C, P, and S), and clock drawing test [32]; and depressive symptom evaluation using an Italian version of the Geriatric Depression Scale (GDS) [33]. The design of the inter-test interval was selected to minimize the risk of bias deriving from practice and recall effects [34]. Observed and reported side effects were obtained by nonspecific querying at the final visit; to ensure full adherence to the study protocol, patients’ compliance was monitored with weekly phone calls and a pill count at the end of the study.

### 2.4. Statistical Analysis

After quality check, the data underwent statistical analysis. Priority sample size estimation was calculated using G*Power 3.1.9.2. software [35]. By assuming an effect size of 0.8, a significance level of 0.05 with a power of 0.80, a minimal sample size of 12 was determined. With the considered dropout approximated to be 20%, a final sample size of 15 was selected. Because of the reduced sample size, the non-normality of the sample distribution was assumed, and, consequently, nonparametric tests were chosen for hypothesis testing. Continuous data were expressed as mean ± standard deviation (StD). The results of cognitive tests before and after treatment were compared for significant differences using Wilcoxon’s signed-rank test to account for paired samples. Significance levels were corrected using Bonferroni’s adjustment for multiple comparisons, resulting in a significance level of *p* < 0.0045. Cohen’s d statistic was also provided to represent the magnitude of the treatment effect. Statistical analysis was performed using SPSS v16.0 software (SPSS Inc., Chicago, IL, USA).

## 3. Results

A total of 15 elder subjects (8 men, 7 women) were recruited. Patients’ mean age at recruitment was 84.5 ± 5.77. After checking for exclusion criteria, all patients were included in the study. Fourteen patients completed the study (93% completion rate); the only premature dropout was due to reported side effects (gastrointestinal discomfort), which promptly regressed after treatment suspension.

Table 1 reports the baseline and final scores of all the neuropsychological measures considered. At baseline, the average MMSE score was 24.21 (StD = 3.09), with some subjects under the threshold for mild cognitive decline. The GDSE average score was 11.10 (StD = 6.08), again denoting, in many subjects, suggestive scores for mild to moderate depressive symptoms. The baseline cognitive subtests also retrieved borderline average values. No statistically significant differences both in the cognitive scores and in the depressive symptom scale emerged at the end of the treatment (*p* > 0.0045).

## 4. Discussion

To the best of our knowledge, this is the first study evaluating the effects of ALA administration on cognition and mood symptoms in a subclinical sample, i.e., patients with nonclinically relevant, possibly age-related cognitive decline but without meeting the diagnostic criteria for major neurocognitive disorder [2]. In our uncontrolled, open-label trial, we found that ALA administration was not associated with substantial improvements in mood, cognitive function, or executive function. While the absence of improvement in neuropsychological subscales may be partly justified by the reduced sample size, we failed at finding differences in the global scores of cognitive tests (such as MMSE) as well as depressive symptoms.

Many in vitro and animal studies have suggested a potential role for the dietary supplementation of ALA in improving memory and mood by reducing oxidative stress effects in the aging brain [21,26,36]. Nevertheless, the effectiveness of ALA as a neuroprotective treatment in human subjects in vivo is still a matter of debate. In patients with diagnosed AD, the administration of ALA 3 months after a standard treatment with acetylcholinesterase inhibitors showed no significant differences in cognitive scores after a 1-year follow-up [27], but a study from the same group suggested that ALA could promote a slower disease progression rate at a 48-month interval [28]. Another preliminary investigation involving the administration of ALA, as well as other antioxidant molecules, paired with antidepressants and lifestyle intervention, resulted in improved cognitive performance in demented patients with depression; however, the results may have been confounded by the concomitant effect on mood and depressive symptoms, which are known to affect cognitive performance, and were not evaluated after the treatment [29]. On the other hand, the few existing randomized controlled trials did not find consistent evidence favoring ALA administration in patients with AD. A small, three-arm trial evaluating the 900 mg/day oral administration of ALA plus vitamins E/C versus coenzyme Q and placebo found that the vitamins E/C–ALA group had a faster cognitive decline compared with placebo [30]; on the other hand, another study comparing omega-3 fatty acid+ALA versus omega-3 alone and placebo found a generally slower trend in cognitive decline in the omega-3+ALA group [37]. While our findings are in line with studies suggesting that further evidence is needed to ascertain the role of ALA as a neuroprotective supplement against cognitive decline, it should be kept in mind that our work is barely comparable with these existing studies both because it is the first study evaluating the cognitive changes in a nonclinical population and ALA was administered in monotherapy.

The mechanisms by which ALA administration is hypothesized to improve cognitive functions are manifold. In vitro investigations demonstrated that dihydrolipoic acid, the reduced form of ALA, acts as an activator of choline-acetyltransferase, a crucial enzyme in the biochemical pathway of acetylcholine. Preclinical investigations have demonstrated the direct effects of short-term ALA administration on cholinergic functions in elder animals [13,38], which could be reflected in improved cognition. In addition, it has been proposed that ALA could act on mood symptoms by upregulating neurotrophic factors, such as the brain-derived neurotrophic factor (BDNF), in animal models of depression [36,39], and this action on mood could, in turn, have an impact on cognitive performance.

Aside from the antioxidant and pro-cognitive effects described in the brain, ALA may exert systemic effects; at the cellular level, ALA alters the nuclear factor kappa-beta related signal transduction cascade, which, in turn, regulates the expression of pro-inflammatory cytokines, metalloproteases, and cell adhesion molecules resulting in an anti-inflammatory effect [40,41]. Plus, ALA and its active metabolite DHLA can both exert a strong chelating effect on redox-active metal ions, such as zinc, copper, iron, and magnesium: this may be relevant as metal ion accumulation constitutes a molecular hallmark of aging processes [42,43]. Finally, the beneficial effects of ALA in lipids and glucose metabolism are relatively well-known [6]. It is possible that many of these effects, which may still result in benefits for mood and memory in elder patients, were not observed in the relatively short follow-up period employed in this study.

It is possible that some of the differences observed between preclinical in vitro and in vivo investigation and clinical research may be explained by cross-species differences in ALA bioavailability and pharmacokinetics. Structurally analogue to the molecules of the vitamin B family, ALA molecules are generally assumed as dietary supplementation in the form of a racemic mixture (1:1 of R-ALA and S-ALA), as S-ALA increases the bioavailability by reducing the polymerization of R-ALA [36,44,45]. Once assumed, ALA undergoes extensive liver metabolism including beta-oxidation [18,46], while it is mostly excreted in urines [47]. It has been demonstrated that ALA has a very short plasmatic half-life (approximately 30 min) and undergoes considerable presystemic elimination; approximately 30% of the oral intake is effectively bioavailable. In addition, there is high variability in its gastrointestinal absorption, ranging between 20% and 40% of the oral intake [48]; moreover, it is possible that the absorption of ALA is in competition with that of other nutrients [18]. Aging commonly reduces the bioavailability of certain nutrients [49], and the impact of antioxidant molecule absorption on the nturients’ beneficial effects in older humans is a still unaddressed topic. This may be particularly relevant as ALA gastrointestinal absorption has been shown to be accelerated by the low-pH environment [50]; while recent investigations suggest that age by itself may have a minor effect in decreasing gastric acid secretion [51], aging increases the prevalence of atrophic gastritis, *Helicobacter pylori* infection, and other conditions that may result in decreased gastric pH [52].

Moreover, while our results speak against a direct role of ALA in improving memory or mood in elder subjects without a diagnosis of AD, it is possible that it still may exert neuroprotective effects that require a longer follow-up to be observed. In addition, our findings need to be interpreted with care as they may be affected by several limitations, including the open design, the small sample size, and the lack of a control group. Taken together, our results underline that further longer duration and/or controlled studies with tightened inclusion criteria are needed to draw firm conclusions about the benefits of ALA supplementation on mood and cognition in elder people.

## 5. Conclusions

Taken together, our results underline that further evidence is needed to draw firm conclusions about the benefits of ALA supplementation on mood and cognition in elder people.

## Figures and Tables

**Table 1:** Cognitive, executive, and mood symptom assessment in patients receiving ALA at baseline (week 0) versus end of study (week 12). ENB2: Esame Neuropsicologico Breve; MMSE: Mini Mental State Examination; GDS: Geriatric Depression Scale; StD: Standard deviation.

	Baseline (T0)		Week 12 (T1)		Wilcoxon Test T0 vs. T1		Effect Size (Cohen’s d)
(Normal Range)	Mean	StD	Mean	StD	z	*p*	d
ENB2-Digit Span (>4)	5.00	1.15	4.90	0.88	−0.45	0.655	0.12
ENB2-Immediated prose memory (>4)	5.80	6.21	6.40	5.13	−0.36	0.719	0.13
ENB2-Delayed prose memory (>6)	7.20	7.64	6.40	6.52	−0.85	0.395	0.14
ENB2-Prose memory with interference 10″ (>1)	2.10	2.02	2.10	2.85	0.00	1.000	0.00
ENB2-Prose memory with interference 30″ (>1)	0.60	1.26	0.60	1.26	0.00	1.000	0.00
ENB2-Trail Making Test A (<202)	355.70″	349.59″	235.00″	272.87″	−1.56	0.110	0.48
ENB2-Phonemic fluency (>3)	6.77	4.19	7.20	3.92	−1.38	0.168	0.13
ENB2-Tangled Figures Test (>9)	13.70	5.12	12.80	4.76	−0.77	0.440	0.23
ENB2-Clock Drawing Test (>3)	3.95	3.37	5.35	3.69	−1.36	0.173	0.49
MMSE (>24)	24.21	3.09	23.15	3.12	−1.13	0.259	0.42
GDS (<11)	11.10	6.08	11.90	5.13	−0.66	0.510	0.18

## Data Availability

The data are kept by the authors who reserve the right to make them available for possible replications of the study.
